# Functional Phenotype of Synovial Monocytes Modulating Inflammatory T-Cell Responses in Rheumatoid Arthritis (RA)

**DOI:** 10.1371/journal.pone.0109775

**Published:** 2014-10-17

**Authors:** Bo Ruem Yoon, Su-Jin Yoo, Yeon ho Choi, Yeon-Ho Chung, Jinhyun Kim, In Seol Yoo, Seong Wook Kang, Won-Woo Lee

**Affiliations:** 1 Department of Microbiology and Immunology, Seoul National University College of Medicine, Seoul, Korea; 2 Department of Internal Medicine, Chungnam National University School of Medicine, Daejon, Korea; 3 Department of Biomedical Sciences, Seoul National University College of Medicine, Seoul, Korea; 4 Cancer Research Institute, Seoul National University, Seoul, Korea; 5 Ischemic/Hypoxic Disease Institute, and Institute of Infectious Diseases, Seoul National University College of Medicine, Seoul, Korea; KAIST, Graduate School of Medical Science & Engineering, Republic of Korea

## Abstract

Monocytes function as crucial innate effectors in the pathogenesis of chronic inflammatory diseases, including autoimmunity, as well as in the inflammatory response against infectious pathogens. Human monocytes are heterogeneous and can be classified into three distinct subsets based on CD14 and CD16 expression. Although accumulating evidence suggests distinct functions of monocyte subsets in inflammatory conditions, their pathogenic roles in autoimmune diseases remain unclear. Thus, we investigated the phenotypic and functional characteristics of monocytes derived from synovial fluid and peripheral blood in RA patients in order to explore the pathogenic roles of these cells. In RA patients, CD14^+^CD16^+^, but not CD14^dim^CD16^+^, monocytes are predominantly expanded in synovial fluid and, to a lesser degree, in peripheral blood. Expression of co-signaling molecules of the B7 family, specifically CD80 and CD276, was markedly elevated on synovial monocytes, while peripheral monocytes of RA and healthy controls did not express these molecules without stimulation. To explore how synovial monocytes might gain these unique properties in the inflammatory milieu of the synovial fluid, peripheral monocytes were exposed to various stimuli. CD16 expression on CD14^+^ monocytes was clearly induced by TGF-β, although co-treatment with IL-1β, TNF-α, or IL-6 did not result in any additive effects. In contrast, TLR stimulation with LPS or zymosan significantly downregulated CD16 expression such that the CD14^+^CD16^+^ monocyte subset could not be identified. Furthermore, treatment of monocytes with IFN-γ resulted in the induction of CD80 and HLA-DR expression even in the presence of TGF-β. An *in vitro* assay clearly showed that synovial monocytes possess the unique capability to promote Th1 as well as Th17 responses of autologous peripheral CD4 memory T cells. Our findings suggest that the cytokine milieu of the synovial fluid shapes the unique features of synovial monocytes as well as their cardinal role in shaping inflammatory T-cell responses in RA.

## Introduction

Monocytes are circulating mononuclear phagocytes that have been generally regarded as systemic precursors for tissue macrophages and inflammatory dendritic cells (DCs) [1]–[3]. Besides their primary role as a precursor, monocytes function as important innate effectors against pathogens through phagocytosis, production of reactive oxygen species (ROS), and secretion of proinflammatory cytokines [4]. It is clear that monocytes are also involved in the pathogenesis of many chronic inflammatory diseases such as RA, Crohn’s diseases, and atherosclerosis [5].

Circulating monocytes are highly plastic and heterogeneous [6]–[9]. In humans, differential expression of CD14 (LPS co-receptor) and CD16 (FcγIII receptor) allows monocytes to be divided into at least two major subsets that have distinct functional activities. The CD14^+^CD16^−^ monocytes, which are referred to as ‘*classical*’ monocytes, are the most prevalent subset representing 80–90% of blood monocytes in a healthy individual. In contrast, CD16^+^ monocytes comprise a relatively smaller subset in circulating human monocytes but are markedly expanded during infection and inflammatory conditions. Thus, they are generally referred to as ‘*proinflammatory’* or *‘nonclassical*’ monocytes. Recent studies demonstrated that CD16^+^ monocytes could be further divided into CD14^+^CD16^+^ and CD14^dim^CD16^+^ subsets. Of note, these two subsets differ in their gene signatures and capacity to secrete inflammatory cytokines in response to external stimuli [8]. CD14^+^CD16^+^ monocytes share many features of CD14^+^CD16^−^ monocytes including a high capacity for phagocytosis and proinflammatory cytokine production in response to TLR ligands. In comparison, CD14^dim^CD16^+^ monocytes exhibit a reduced capacity for phagocytosis and produce less ROS, but have the unique ability to patrol the blood vessel endothelium for signs of damage and infection. Since perturbation of monocyte subsets, especially significant expansion of CD16^+^ monocytes, is seen in many chronic inflammatory conditions, it has been suggested that CD16^+^ monocytes play a pivotal role in the pathophysiology of many autoimmune diseases.

RA is a systemic and chronic autoimmune disease that primarily targets synovial membranes [10]. Despite extensive studies, the cause of RA is still unknown although a complex interplay among genetic factors and environmental triggers are thought to be involved [11]. The presence of autoantibodies generated via T-cell-dependent processes in RA patients and mouse models underscore the importance of adaptive immunity as the central contributor to early pathogenesis [11]; however, a growing body of evidence has revealed that a variety innate effector cells, such as monocytes/macrophage, are also closely involved in the development of synovial inflammation in RA [10]. In particular, massive infiltration of activated monocytes/macrophages are frequently observed in synovial membranes of RA patients where they are a major source of many cytokines including TNF-α, IL-1β, IL-8, and GM-CSF in the inflamed joints. Of note, accumulating monocytes in synovial tissue of RA patients express significantly increased CD16 on their surface and produce enormous amounts of TNF-α in response to TLR stimulation [12], [13].

Given the presence of monocytes at the sites of both T-cell priming and antigen entry, their possible roles in the regulation of T-cell responses have been suggested [7]. Indeed, it has become clear from recent studies that inflammatory monocytes influence T cell polarization and expansion and shape T-cell responses [7], [14]. Recently, *in vivo* activated-monocytes derived from the inflamed joint fluid of active RA patients have been shown to specifically promote pathogenic T-cell responses, mainly in a cell contact-dependent manner [15]. In this context, monocytes are not just producers of inflammatory cytokines, but are closely linked to the modulation of adaptive immune responses, especially T-cell responses, in autoimmune diseases [7], [15], [16].

In the present study, we sought to investigate the phenotypic and functional characteristics of monocytes derived from synovial fluid and peripheral blood of RA patients in order to further explore their pathogenic roles. Here we found that CD14^+^CD16^+^, but not CD14^dim^CD16^+^, monocytes were predominantly expanded in synovial fluid and to a lesser degree in peripheral blood of RA patients. Further, the synovial monocytes differentially expressed co-signaling molecules such as CD80 and CD276, suggesting distinct regulatory roles for T-cell responses. Our findings also suggest the possibility that the cytokine milieu of the synovial fluid confers unique phenotypic features to synovial monocytes and hence, shapes their roles in directing the inflammatory T-cell response during RA.

## Materials and Methods

### Cell preparation

The study protocols were reviewed and approved by the institutional review board of Chungnam National University Hospital (IRB No. 2012-01-024) and the institutional review board of Seoul National University Hospital (IRB No.1109-055-378). All RA patients and healthy volunteers provided their written informed consent to participate in this study. Peripheral blood and synovial fluid of RA patients were collected after obtaining the written informed consent at Department of Internal Medicine, Chungnam National University Hospital. Peripheral blood of healthy volunteers was drawn after obtaining the written informed consent at Seoul National University College of Medicine. Mononuclear cells were isolated from peripheral blood and synovial fluid by density gradient centrifugation (Bicoll separating solution; BIOCHROM Inc, Cambridge, UK). Monocytes were positively separated from peripheral blood mononuclear cells (PBMC) and synovial fluid mononuclear cells (SFMC) with anti-CD14 microbeads (Miltenyi Biotec Inc, Auburn, CA). In some experiments, CD14^+^CD16^−^ monocytes were positively purified using anti-CD14^+^ microbeads from PBMC in which CD16^+^ monocyte were depleted by CD16 monocyte isolation kit (Miltenyi Biotec Inc). CD4 memory T cells were negatively isolated from PBMC with human memory CD4^+^ T cell enrichment kit (EasySep negative selection; STEMCELL technologies Inc, Vancouver, Canada).

### Flow cytometric analysis

PBMC and SFMC were stained for 30 min at 4 degree with the following antibodies: APC-CD3, PE-Cy5-CD4, APC-Cy7-CD14, PE-Cy5-CD16, APC-CD19, APC-CD56, PE-CD80, PE-CD86, APC-CD276, FITC-HLA-DR (all from BD Bioscience, San Jose, CA), PE-CX_3_CR1 (eBioscience, San Diego, CA), and PE-CCR2 (R&D systems, Minneapolis, MN). Stained cells were acquired by a BD LSRII (BD bioscience) and analyzed by using Flowjo software (Tree star, Ashland, OR).

### Cell culture

Purified monocytes and CD4 memory T cells were cultured in RPMI 1640 medium supplemented with 10% fetal bovine serum (FBS), 1% penicillin/streptomycin (penicillin 100units/ml and streptomycin 100 ug/ml) and 1% L-glutamine (2 mM). For T cell-monocyte coculture, purified monocytes were seeded at 5×10^3^ into each well of U-bottomed 96-well plate and incubated with soluble anti-CD3 and anti-CD28 antibodies (1 µg/ml of each; BD bioscience) in the presence of 100ng/ml LPS (Sigma-Aldrich Inc, St. Louis, MO). After incubation for 1 hour, 2.5×10^4^ CD4^+^ memory T cells were added into each well and co-cultured with LPS-activated monocytes for 7 days. In some experiments, purified monocytes were treated for 18 hours with TGF-β (10 ng/ml; R&D systems) in the presence or absence of the indicated recombinant human cytokines such as IL-1β, IL-6 (25 ng/ml of each; both from R&D system), TNF-α (25 ng/ml; ProSpec, East Brunswick, NJ) and IFN-γ (25 ng/ml; eBioscience) and SB431542 (TGF-β signaling inhibitor; Calbiochem, La Jolla, CA), After 18 hour-treatment, the monocytes were stained with the following antibodies: APC-Cy7-CD14, PE-CD16, PE-CD80, and FITC-HLA-DR. The stained cells were acquired by a BD LSRII (BD bioscience).

### Intracellular cytokine staining (ICS) and enzyme-linked immunosorbent assay (ELISA)

For intracellular cytokine staining, CD4 T cells co-cultured with monocytes for 7 days were re-stimulated 6 hours with 50ng/ml PMA (Sigma-Aldrich Corp. St. Louis, MO) and 1 ug/ml ionomycin (Sigma-Aldrich) in the presence with Golgiplug (BD Bioscience) for last 4 hours. The cells were fixed and permeablized with BD Cytofix/Cytoperm kit and stained with the following antibodies: PE-IL-17A (eBioscience), PE-Cy7-IFN-γ, and PE-Cy5-CD4 (both from BD Biosciences), followed by analysis using a BD LSRII. In some experiments, intracellular cytokine staining was performed with freshly isolated PBMC and SFMC from RA patients. The amount of IL-17A and IFN-γ in coculture supernatant was quantified by commercial ELISA kits (eBioscience for IL-17A ELISA kit and BioLegend for IFN-γ ELISA kit). The measurement of OD (Optical density) was performed using DTX 880/multimode detector (Beckman coulter, Brea, CA).

### Quantitative RT-PCR

Total RNA was extracted from freshly isolated or cocultured cells using TRIzol reagents (life technologies, Grand Island, NY) and cDNA was synthesized by GoScript reverse transcription system (Promega, Madison, WI). Real-time quantitative RT-PCR was performed in triplicates on a 7500 PCR system (Applied Biosystems, Grand Island, NY) using following primers: *CD16*∶5′-GCTCCGGATATCTTTGGTGA-3′ and 5′-CTCCCTGGCACTTCAGAGTC-3′; *CD68*∶5′-ACTGAACCCCAACAAAACCA-3′ and 5′-TTGTACTCCACCGCCATGTA3′; *CD80*∶5′-GGGAAAGTGTACGCCCTGTA-3′ and 5′-GCTACTTCTGTGCCCACCAT-3′; *CD86*∶5′-TGGAACCAACACAATGGAGA-3′and 5′-GGTTGCCCAGGAACTTACAA-3′; *CX_3_CR1*∶5′-GCAAGAAGCCCAAGAGTGTC-3′ and 5′-ATGCTGATGACGGTGATGAA-3′; *CD276*∶5′-ACCATCACACCCCAGAGAAG-3′ and 5′-GCCAGATGAGGTTGAGCTGT-3′; *CCR2*∶5′-CTGTCCACATCTCGTTCTCGGTTTA-3′ and 5′-CTGAACTTCTCCCCAACGAA-3′; *CCR5*; 5′-CTGAGACATCCGTTCCCCTA-3′ and 5′-TTGCCCACAAAACCAAAGAT-3′; *TLR2*∶5′-ATTGTGCCCATTGCTCTTTC-3′and 5′-CTGCCCTTGCAGATACCATT-3′; *TLR4*∶5′-TCTTCAACCAGACCTCTACATTCCA-3′ and 5′-GGAACATCCAGA GTGACATCACAG-3′; *TLR7*∶5′-TCACTCCATGCCATCAAGAA-3′ and 5′-ACCATCTAGCCCCAAGGAGT-3′; *TLR8*∶5′-TTTTCTTCATTGGGCCAAAC-3′ and 5′-GAATGGCTGAAA ATTCAGTTCC-3′; *β-actin*: 5′-GGACTTCGAGCAAGAGATGG-3′and 5′-AGCACTGTGTTGGCGTACAG-3′. The levels of gene expression were normalized to the expression of *β-actin*. The comparative C_T_ method (ΔΔC_T_) was used for the quantification of gene expression.

### Statistical analysis

Two-tailed paired *t*-test or unpaired *t*-test was done to analyze data using Prism 5 software (GraphPad Software Inc, La Jolla, CA) as indicated in the figure legends. *P* values of less than 0.05 were considered statistically significant.

## Results

### CD14^+^CD16^+^, but not CD14^dim^CD16^+^, monocytes are predominantly expanded in RA patients

**Figure 1:**
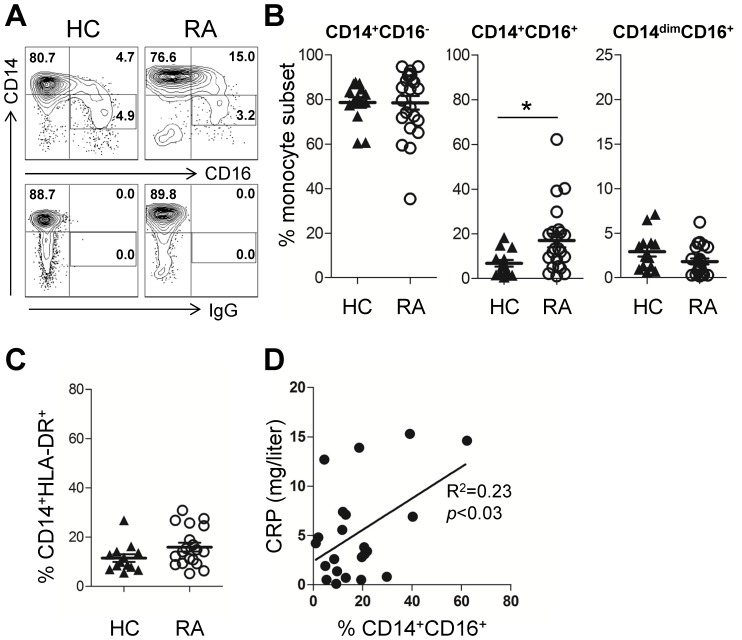
Frequency of CD14^+^CD16^+^, but not CD14^dim^CD16^+^, monocytes is increased in peripheral blood of rheumatoid arthritis (RA) patients compared with healthy controls (HC). (**A**) Representative contour plot of peripheral monocytes from HC (left) and RA patients (right). Monocytes were defined as cells expressing HLA-DR but not expressing any lineage markers, such as CD3 (T cells), CD19 (B cells), or CD56 (NK cells). Gated cells were subdivided into three monocyte subsets by their expression of CD14 and CD16. Number in rectangular gate indicates the frequency of CD14^dim^CD16^+^ monocytes. (**B**) Frequencies (%) of three monocyte subsets in peripheral blood from HC (n = 14) and RA patients (n = 23) (**C**) Frequencies (%) of monocytes expressing CD14 and HLA-DR among peripheral blood mononuclear cells (PBMC) between HC and RA patients. (**D**) The frequency of peripheral CD14^+^CD16^+^ monocyte was plotted against serum C-reactive protein levels (CRP) among RA patients (n = 22). Each data point represents an individual subject; horizontal bars and error bars show the mean ± SEM. * = *P<*0.05 by unpaired *t*-test (B). *P* value in (D) was obtained using the Pearson correlation analysis.

Perturbation in the proportions of circulating monocyte subsets has been observed in many inflammatory conditions [9], [13]. We first examined whether the frequency of different monocyte subsets was changed in RA patients compared to healthy controls. Based on the phenotypic definition of human monocytes in previous reports [4], [9], CD14 and CD16 expression on peripheral blood monocytes (PBMO) from healthy controls and RA patients were analyzed. For this study, monocytes were defined as cells expressing the pan-monocyte marker, HLA-DR, but not bearing any B, T, or NK cell lineage markers in order to avoid contamination with HLA-DR or CD16 expressing B or NK cells, respectively. In agreement with previous reports [4], [9], CD16^+^ monocytes were further divided into two subsets, “intermediate” CD14^+^CD16^+^ and “nonclassical” or “patrolling” CD14^dim^CD16^+^ monocytes, by their CD14 expression level (Fig. 1A). As shown in the Figure 1B, the frequency of CD14^+^CD16^+^ monocytes was significantly enhanced in the peripheral blood of RA patients compared with age-matched healthy controls (Mean ± S.E.M., 16.96±3.05% vs. 7.02±1.50%, *p* = 0.0207). However, the frequency of peripheral CD14^dim^CD16^+^ monocytes did not significantly differ between RA patients and healthy controls, (2.92±0.54% vs. 1.81±0.33%). Although both monocyte subsets express CD16, these subsets are considered phenotypically and functionally distinct [4], [9]. Consequently, the ratio of CD14^+^CD16^+^ to CD14^dim^CD16^+^ monocytes in RA patients was six times higher than that in healthy controls (21.83±5.49 fold vs. 3.54±0.94 fold), indicating the selective expansion of CD14^+^CD16^+^ monocytes in the peripheral blood of RA patients. However, the frequency of monocytes, defined as HLA-DR^+^CD14^+^, among PBMCs did not differ between RA patients and healthy controls (Fig. 1C). To examine clinical relevance of CD14^+^CD16^+^ monocyte, we analyzed the association between the frequency of CD14^+^CD16^+^ monocyte and clinical parameters in the patient. As seen in Figure 1D, the frequency of peripheral CD14^+^CD16^+^ monocytes was significantly correlated with disease activity measured by serum C-reactive protein levels among the RA patients (Fig. 1D).

**Figure 2:**
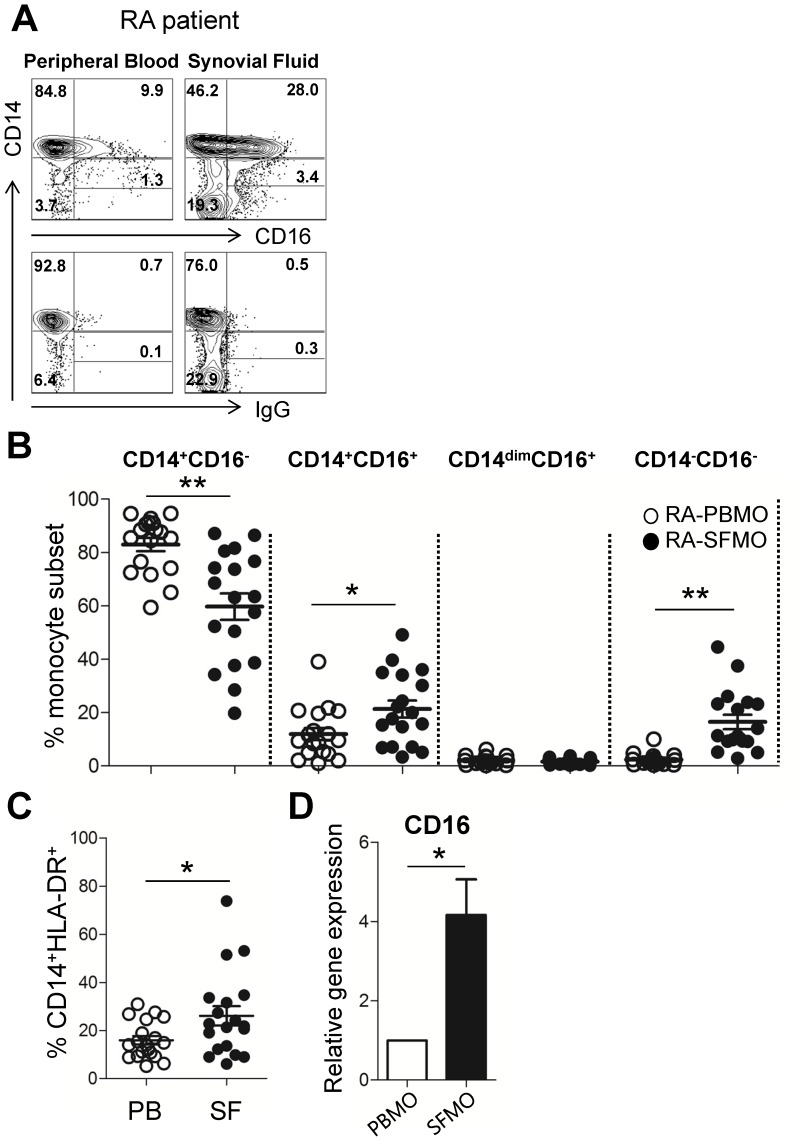
CD14^+^CD16^+^ monocytes are more expanded in synovial fluid (SF), which is the site of inflammation in RA patients. (**A**) Representative contour plot of CD14 and CD16 expression on paired peripheral (left) and synovial monocytes (right) from RA patients. Number in rectangular gate indicates the frequency of CD14^dim^CD16^+^ monocytes. (**B**) Frequencies (%) of different monocyte subsets which were subdivided by differential expression of CD14 and CD16, in paired peripheral blood and synovial fluid RA patient samples (n = 18) (**C**) Frequencies (%) of CD14^+^HLA-DR^+^ monocytes in peripheral blood and synovial fluid derived from the same RA patients (n = 18) (**D**) Relative gene expression of CD16 in monocytes purified from peripheral blood and synovial fluid of the same RA patients (n = 5). Each data point represents an individual subject; horizontal bars and error bars show the mean ± SEM. * = *P<*0.05; ** = *P<*0.01 by paired *t*-test in (**B and C**). Bars show the mean ± SEM. * = *P<*0.05 by paired *t*-test in (**D**). PBMO (peripheral blood monocytes) and SFMO (synovial fluid monocytes).

We next investigated whether the CD14^+^CD16^+^ monocyte population was more expanded in synovial fluid, which is the site of inflammation in RA patients (Fig. 2). To this end, monocyte subsets were compared between the periphery and synovial fluids, which were prepared simultaneously from the same RA patients. In synovial fluid, the percentage of classical CD14^+^CD16^−^ monocytes was significantly reduced compared to that of the peripheral blood of the same patients (Fig. 2B, 59.76±4.95% vs. 83.06±2.48%; *p*<0.0005). Conversely, there was a significant increase of CD14^−^CD16^−^HLA-DR^+^ cells as well as CD14^+^CD16^+^ monocytes in synovial fluid, while CD14^dim^CD16^+^ monocytes were only minimally changed. In 15 out of 18 patients tested, CD14^+^CD16^+^ monocytes were increased more than two fold in synovial fluid when compared with peripheral blood (21.33±3.18% vs. 11.97±2.25%; *p*<0.05). We also revealed that the frequency of HLA-DR^+^CD14^+^ monocytes, which includes CD14^+^CD16^+^ monocytes, is significantly increased in synovial fluid (*p*<0.05), suggesting that the absolute number of CD14^+^CD16^+^ monocytes is also likely increased in synovial fluid (Fig. 2C). Enhanced CD16 expression in synovial monocyte was also confirmed at the level of gene expression and found to be significantly higher in CD14^+^ monocytes purified from synovial fluids than those purified from peripheral blood of the same RA patient (Fig. 2D).

### Co-signaling molecules are differentially expressed in synovial and peripheral monocytes in RA patients

*In vivo* activated monocytes derived from the synovial fluid of RA patients play a critical role in inducing pathogenic CD4 T-cell responses in a cell contact-dependent manner [15]. Thus, the differential expression of surface molecules by synovial monocytes may be responsible for this finding. To explore a pathogenic role of synovial monocytes in RA patients, we evaluated the expression of major co-signaling molecules on monocytes freshly prepared from paired peripheral blood and synovial fluid samples in RA.

**Figure 3:**
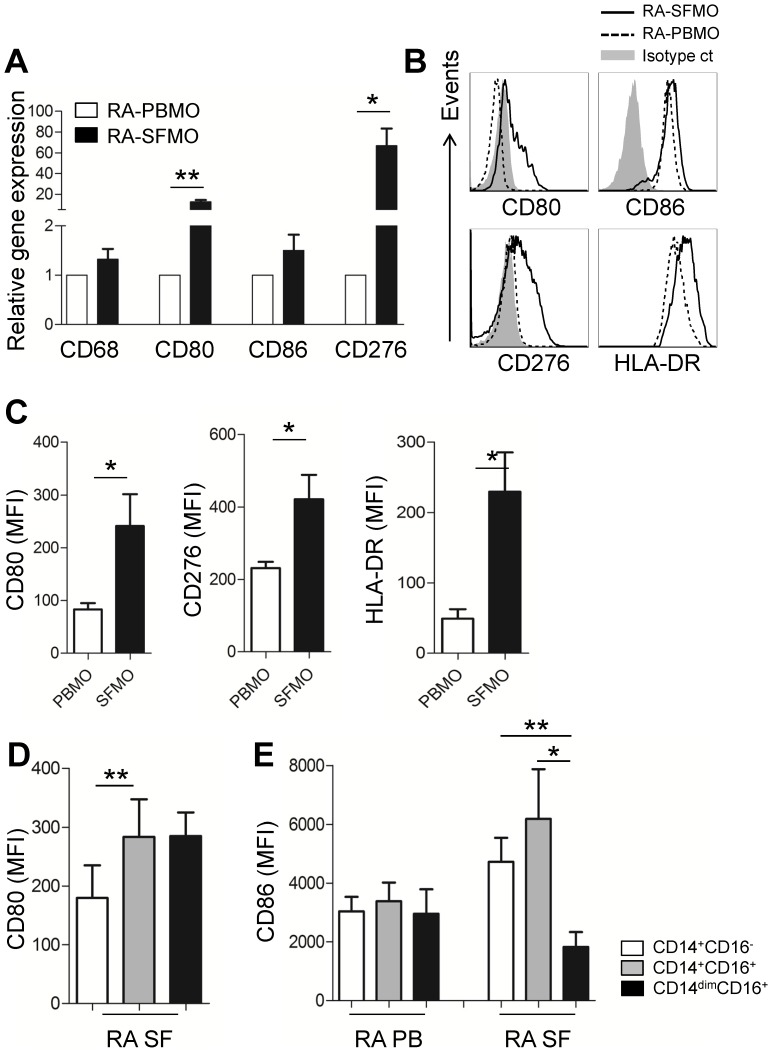
RA patient-derived synovial monocytes predominantly express B7 family member co-signaling molecules, CD80 and CD276. (**A**) Quantitative RT-PCR analysis of CD68, CD80, CD86, and CD276 gene expression in paired peripheral and synovial monocytes from RA patients (n = 4 or 5). (**B**) Representative flow cytometric analysis of CD80, CD86, CD276, and HLA-DR expression on paired peripheral and synovial monocytes from RA patients (solid line: synovial monocytes; dashed line: peripheral monocytes; gray shaded: isotype control). (**C**) Surface expression of CD80, CD276, and HLA-DR on peripheral and synovial monocytes from RA patients (n = 7 or 12). (**D**) Expression of CD80 on three different monocyte subsets of synovial monocytes from RA patients (n = 8). (**E**) Expression of CD86 on three different monocyte subsets of synovial and peripheral monocytes from RA patients (n = 8). Bars show the mean ± SEM. * = *P<*0.05; ** = *P<*0.01 by paired *t*-test in (**A, D, and E**) and unpaired *t*-test (**C**). MFI means mean fluorescent intensity. PBMO (peripheral blood monocytes) and SFMO (synovial fluid monocytes).

Among the molecules tested, the most prominent differences were observed in the expression of CD80 (B7.1) and CD276 (B7–H3), which are members of B7 co-signaling family [17], between synovial and peripheral monocytes in RA patients (Fig. 3). Expression of CD80 and CD276 mRNAs in purified synovial monocytes were drastically upregulated (Mean ± S.E.M., 12.52±1.91 folds and 66.67±16.68 folds, respectively) when compared with peripheral monocytes from the same RA patient. In contrast, expression of CD86, another B7 co-stimulatory molecule, and CD68, a marker of tissue macrophages, were not changed in synovial monocytes (1.50±0.32 and 1.33±0.21 fold, respectively. Not significant) (Fig. 3A). Furthermore, peripheral monocytes from both healthy controls (data not shown) and RA patients constitutively express CD86 at high levels, but do not express CD80 or CD276 (Fig. 3B). Thus, elevated CD80 and CD276 expression appeared to be a feature unique to synovial monocyte in RA patients, a finding that was confirmed by examining the surface expression of these molecules by flow cytometric analysis (*p*<0.05 and *p*<0.05, respectively; Fig. 3C). HLA-DR is a human MHC class II molecule that complexes with cognate antigen and gives the first signal to the T cell receptor (TCR) on CD4 T cells [18]. In humans, monocytes constitutively expressed HLA-DR at high levels, but HLA-DR expression on synovial monocytes was found to be significantly higher than that of peripheral monocytes (MFI; 4,907±1,132 vs. 17,817±2,917, *p*<0.0001, Fig. 3C). Upregulated expression of CD80, CD276, and HLA-DR by synovial monocytes suggests that these cells differentially regulate T cell responses. When the expressions of CD80 and CD86 were compared among three different monocyte subsets in RA patients, CD80 expression was significantly higher in CD14^+^CD16^+^ monocytes in synovial fluid (Fig. 3D, *p*<0.01), whereas CD86 expression was markedly lower in synovial CD14^dim^CD16^+^ (Fig. 3E, *p*<0.05).

### Differential expression of chemokine receptors and TLRs in synovial and peripheral monocytes of RA patients

**Figure 4:**
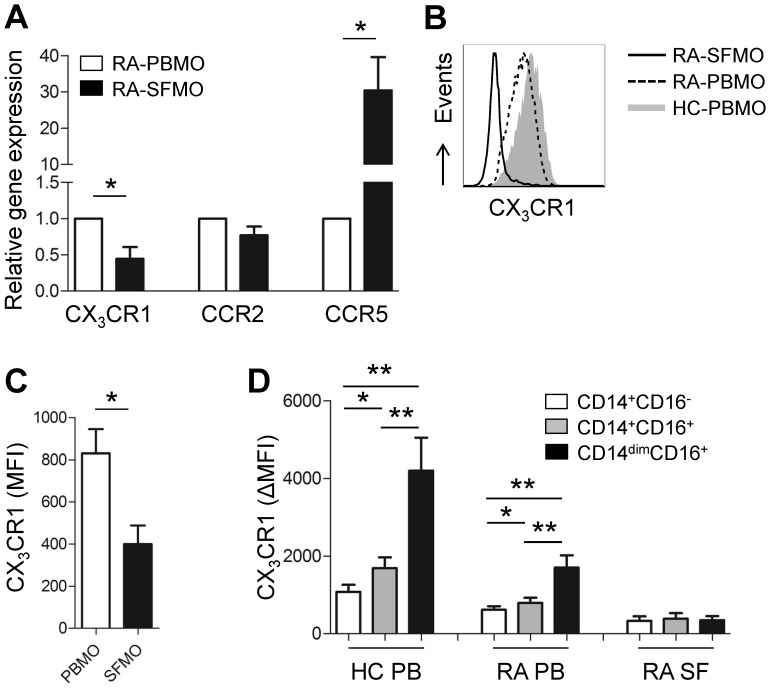
Expression of monocyte subset-specific chemokine receptors differs between peripheral and synovial monocytes from RA patients. (**A**) Quantitative RT-PCR analysis of CX_3_CR1, CCR2, and CCR5 gene expression in paired peripheral and synovial monocytes from RA patients (n = 4 or 5). (**B**) Representative flow cytometric analysis of CX_3_CR1 expression on peripheral monocytes from HC and paired peripheral and synovial monocytes from RA patients (solid line: RA synovial monocytes; dashed line: RA peripheral monocytes; gray shaded: HC peripheral monocytes). (**C**) Flow cytometric analysis of CX_3_CR1 expression on paired peripheral and synovial monocytes from RA patients (n = 8). (**D**) Expression of CX_3_CR1 on three different monocyte subsets of peripheral monocytes from HC (n = 8) and peripheral and synovial monocytes from RA patients (n = 8). Bars show the mean ± SEM. * = *P<*0.05; ** = *P<*0.01 by paired *t*-test (**A, C, and D**). MFI = mean fluorescent intensity.

Chemokines and their receptors are critical for mediating monocyte recruitment into sites of inflammation [6], [19]. Since perturbation of monocyte subsets derived from synovial fluid were clearly seen in RA patients (Fig. 2), we next examined whether the expression of chemokine receptors differ between synovial and peripheral monocytes in the same RA patients. Consistent with previous findings [19], [20], CCR5 gene expression in synovial monocytes was markedly upregulated, by 30.41 fold (±9.19), compared with peripheral monocytes (*p*<0.05). In contrast to CCR5, mRNA expression of CX_3_CR1 was significantly reduced by 50% in synovial monocyte (Fig. 4A). CD16^+^ monocytes are known to express high levels of CX_3_CR1 and low levels of CCR2. In addition, the CX_3_CR1-CX_3_CL1 interaction is critical for efficient trans-endothelial migration of CD16^+^ monocyte [6]. In spite of prominent expansion of CD14^+^CD16^+^ monocyte in RA patients (Fig. 1B and 2B), the expression of CX_3_CR1 was decreased moderately on peripheral CD16^+^ monocytes and more strikingly on synovial CD16^+^ monocytes from RA patients (Fig. 4B and 4C). Of note, CX_3_CR1 expression was almost comparable among the three monocyte subsets in RA synovial monocytes (Fig. 4D). The expression of CCR2, another monocyte subset-specific chemokine receptor, did not differ significantly between healthy controls and RA patients (Fig. 4A).

**Figure 5:**
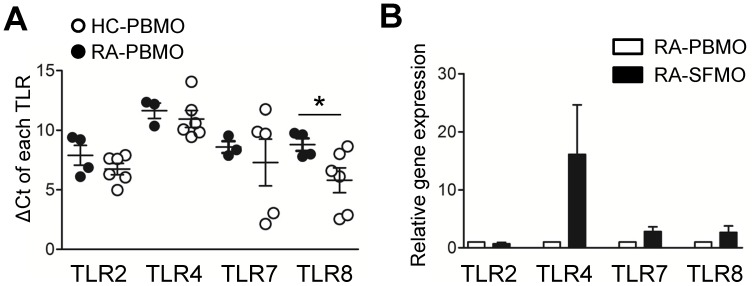
Expression of toll-like receptors differs between peripheral and synovial monocytes derived from RA patients. (**A**) Quantitative RT-PCR analysis of TLR2, 4, 7, and 8 gene expression in peripheral monocytes between HC and RA patients. Expression was normalized to β-actin and ΔCt was calculated by subtracting the Ct of β-actin from the Ct of each TLR gene. Each data point represents an individual subject. (**B**) Comparison of TLR2, 4, 7, and 8 gene expression in paired peripheral and synovial monocytes from RA patients (n = 6); Horizontal bars and error bars show the mean ± SEM. * = *P<*0.05 by unpaired *t*-test with Welch's correction in (**A**).

Several studies have recently demonstrated that TLR-stimulated monocytes are required for optimal induction of Th17 cells, which are involved in the pathogenesis of various autoimmune diseases [15], [16]. Thus, we analyzed TLR expression in monocytes from blood and synovial fluid of RA patients or healthy controls. In peripheral monocytes, quantitative PCR analysis revealed that TLR8 gene expression was markedly elevated in RA patients, whereas TLR2, TLR4, and TLR7 (Fig. 5A) were comparably expressed between RA patients and healthy controls. In monocytes from the same RA patients, the levels of TLR4 gene expression in those derived from synovial fluid was approximately 10 fold greater than in the peripheral monocytes (Fig. 5B).

### Cytokines shape the unique functional phenotype of synovial monocytes

**Figure 6:**
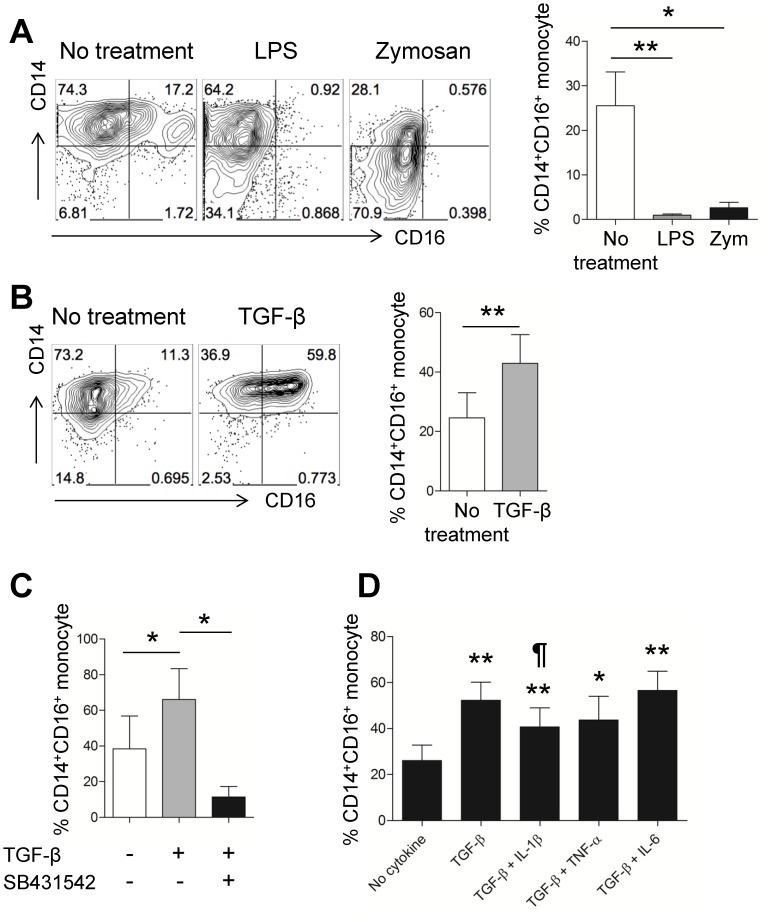
Cytokines shape the unique functional phenotype of synovial monocytes. PBMC or purified monocytes derived from HC were stimulated for 18 hours with the indicated TLR agonists or cytokines and expression of CD14 and CD16 were analyzed by flow cytometry. (**A**) Representative flow cytometric analysis of change in CD16 expression on monocytes (left) and bar graph showing the decreased frequency of the CD14^+^CD16^+^ monocytes after stimulation with LPS (10 ng/ml) or zymosan (Zym; 10 ug/ml) (right; n = 5). (**B**) Representative flow cytometric analysis of change in CD16 expression on monocytes (left) and bar graph showing the increased frequency of CD14^+^CD16^+^ monocytes (right; n = 10) after treatment with recombinant human TGF-β (10 ng/ml). (**C**) Purified CD14^+^CD16^−^ monocytes were treated with TGF-β in the absence or presence of TGF-β signaling blocker, SB431542. TGF-β-induced CD16 expression on monocytes is significantly inhibited by SB431542 (n = 3). (**D**) Frequency of the CD14^+^CD16^+^ monocytes treated with the indicated proinflammatory cytokines [IL-1β (25 ng/ml), IL-6 (25 ng/ml), and TNF-α (25 ng/ml)] in the presence of TGF-β (n = 9). Bars show the mean ± SEM. * = *P<*0.05; ** = *P<*0.01 by Mann Whitney test in (**A**) and paired *t*-test in (**B and C**). * = *P<*0.05; ** = *P<*0.01 versus no cytokine group in (**D**) and ¶ = *P<*0.05 versus TGF-β group by paired *t*-test in (**D**).

**Figure 7:**
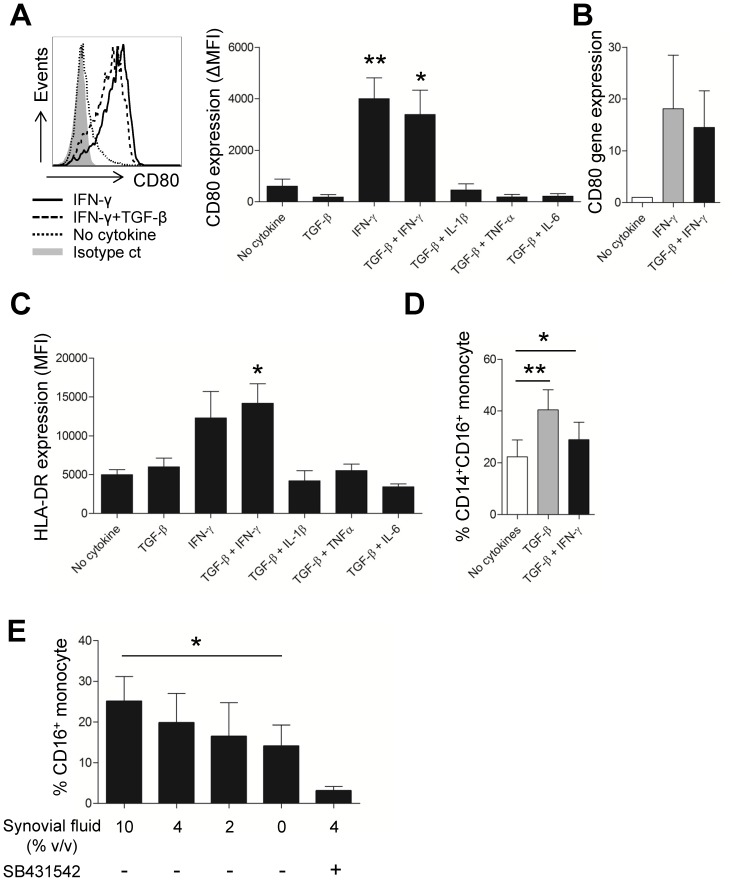
Cytokines are contributing factors for the unique functional phenotypes of RA synovial monocytes. PBMC or purified monocytes derived from HC were stimulated for 18 hours with the indicated cytokines or synovial fluid and expression of indicated molecules were analyzed by flow cytometry (**A**). Representative flow cytometric analysis of CD80 expression on monocytes treated with the indicated cytokines [left; solid line: IFN-γ (25 ng/ml), dashed line: IFN-γ plus TGF-β, dotted line: no cytokine, gray shaded: isotype control]. The change in CD80 expression on monocytes treated with various combinations of cytokines (right; n = 6). MFI, mean fluorescent intensity. (**B**). Quantitative RT-PCR analysis of CD80 gene expression on monocytes after treatment with IFN-γ alone or IFN-γ plus TGF-β (n = 3). (**C**) The change in HLA-DR expression on monocytes treated with various combinations of cytokines (n = 5). (**D**) Frequency of the CD14^+^CD16^+^ monocytes treated with TGF-β alone or IFN-γ plus TGF-β (n = 13). (**E**) Induced CD16 on CD14^+^ monocytes treated for 18 hours with various concentration of synovial fluid derived from RA patients with or without SB431542 (n = 5). Bars show the mean ± SEM. * = *P<*0.05; ** = *P<*0.01 versus no cytokine group in (**A, C, and D**). * = *P<*0.05; versus no treatment group in (**E**).

The expansion of circulating CD16^+^ monocytes has been associated with various acute or chronic inflammatory diseases [21]. We, and others, have shown that CD14^+^CD16^+^, but not CD14^dim^CD16^+^, monocytes are markedly enhanced at sites of inflammation in chronic autoimmune disorders such as RA and Crohn’s diseases (Fig. 2B) [14]. One possible mechanism for this is the *de novo* expression of CD16 on monocytes caused by exposure to the inflammatory milieu of synovial fluid. To test this possibility, purified CD14^+^ monocytes from healthy donor were stimulated with TLR ligands or various cytokines and CD16 expression assessed by flow cytometric analysis. As seen in Figure 6A, the CD14^+^CD16^+^ monocyte subset almost completely disappeared following stimulation with LPS (ligand for TLR4) or zymosan (ligand for TLR2) for 18 hours (*p*<0.01 and *p*<0.05, respectively). In the noninfectious setting of chronic inflammatory diseases such as RA, it is likely that cytokines in synovial fluid contribute to monocyte activation. To this end, we found that TGF-β clearly induced elevated CD16 expression on CD14^+^ monocytes from healthy controls (Fig. 6B). Approximately 20% of monocytes induced CD16 expression on their cell surface following TGF-β treatment and this induction was successfully inhibited by treatment with a selective blocker of TGF-β signaling, SB431542, confirming this effect was due to TGF-β (Fig. 6C). No additive effects of co-treatment with pro-inflammatory cytokine such as IL-1β, TNF-α, or IL-6 were observed in the present experiments, whereas co-treatment with IL-1β induced a weak inhibitory effect on CD16 induction (*p*<0.05) (Fig. 6D). We next explored the cytokine environment in which expression of CD80 and HLA-DR are induced by monocytes. Since TGF-β is a key inducer of CD16 on monocytes, purified monocytes were incubated with various cytokines in the presence of TGF-β. The results clearly revealed that IFN-γ is a potent inducer of CD80 and HLA-DR expression on monocytes (Fig. 7A–D). Furthermore, TGF-β treatment exhibited no significant effect on the expression of CD80 or HLA-DR (Fig. 7A–D). Likewise, CD80 mRNA expression with IFN-γ alone or TGF-β plus IFN-γ treatment was markedly enhanced, by 18.09 fold (±10.36) and 14.53 fold (±7.04), respectively (Fig. 7B). Of note, TGF-β plus IFN-γ treatment significantly induced CD16 expression, although not as much TGF-β treatment alone (Fig. 7D). We finally examined whether RA synovial fluid could up-regulate CD16 expression on monocytes. As seen in Figure 7E, synovial fluid enhanced the expression of CD16 on CD14^+^ monocyte from healthy donor with dose-dependent manner. This up-regulation was greatly inhibited by SB431542, a specific blocker of TGF-β signaling. These findings suggest that the distinct functional phenotype of synovial monocytes seen in RA patients may be induced by the cytokine milieu specific to their synovial fluid.

### Synovial monocytes have a unique capability to promote proinflammatory Th17 and Th1 responses in RA patients

**Figure 8:**
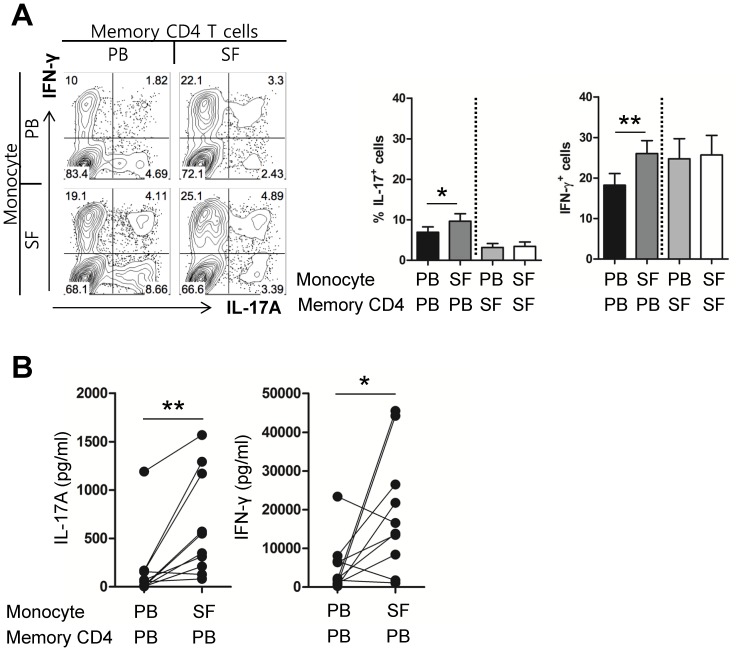
RA patient-derived synovial monocytes have a unique capacity to promote inflammatory Th17 and Th1 responses. Monocytes and CD4^+^ memory T cells were isolated from peripheral blood and synovial fluid of RA patients and were cocultured in a crisscross pattern for 7 days in the presence of soluble anti-CD3 and anti-CD28 antibodies (1 µg/ml of each) and LPS (100 ng/ml) as described in Material and Methods. (**A**) Representative contour plot of ICS (left) and frequency (%) of IL-17A- and IFN-γ-producing CD4 T cells in the cirsscross colculture (right; n = 14). (**B**) The amounts of IL-17A (left) and IFN-γ (right) in the culture supernatant were quantified using conventional ELISA kits (n = 10). Each pair of symbols represents one experiment. Bars show the mean ± SEM. * = *P<*0.05; ** = *P<*0.01 by paired *t*-test in in (**A and B**).

The CD14^+^CD16^+^ monocyte subset is known to be responsible for pathogenesis of chronic inflammatory diseases and therefore, these cells are referred as to as inflammatory monocytes [14]. To examine whether synovial monocytes in RA patients have a unique capability to modulate T-cell responses, we conducted crisscross cocultures using CD4 memory T cells and monocytes from the synovial fluid and peripheral blood of the same RA patients for 7 days in the presence of soluble anti-CD3, anti-CD28 antibodies and LPS (Fig. 8A). On day 7, cytokine production of co-cultured CD4 T cells was analyzed using intracellular cytokine staining and ELISA. As shown in Figure 8A, proportions of IL-17A^+^ and/or IFN^+^ CD4^+^ T cells were significantly higher in peripheral CD4 memory T cells when cultured with synovial monocytes than with peripheral monocytes from the same RA patients. In contrast, induction of IL-17A^+^ and/or IFN-γ^+^ cells from synovial CD4 memory T cells was not affected regardless of monocyte origin in RA patients (Fig. 8A). Consistent with data from the flow cytometric analysis, the amount of IL-17A and IFN-γ in the culture supernatant was significantly increased when peripheral CD4 memory T cells were co-cultured with synovial monocytes (Fig. 8B). Taken together, activated monocytes derived from synovial fluid possess a unique capability to promote production of proinflammatory IL-17 and IFN-γ from peripheral CD4 memory T cells.

## Discussion

In the present study, we demonstrate that proinflammatory CD14^+^CD16^+^, but not CD14^dim^CD16^+,^ monocytes are moderately expanded in peripheral blood and prominently in synovial fluid of RA patients compared to healthy controls. Synovial monocytes have a unique B7 family co-signaling molecule and chemokine receptor expression profile and these distinct functional phenotypes could be induced under the specific cytokine milieu within the synovial fluid. Moreover, RA patient synovial monocytes, but not peripheral monocytes, possess the capability to promote pathogenic Th17 and Th1 responses from autologous peripheral CD4 memory T cells *in vitro*.

One pathologic hallmark of RA is the massive infiltration of activated monocytes/macrophages into inflamed joints where these cells are the primary producers of TNF-α, the major pro-inflammatory cytokine in RA pathogenesis [22]. In addition, activated monocytes from synovial fluid of RA patients function as antigen presenting cells to promote pathogenic CD4 T cell responses at sites of inflammation [7], [15], [16]. Therefore, monocytes/macrophages have become a major target in the treatment of RA. Given that peripheral CD16^+^ monocytes are markedly expanded in active RA patients, and their levels correlate with disease severity and responsiveness to therapy, these cells have been implicated in the pathophysiology of RA [19].

Our data clearly reveal that the expansion of peripheral and synovial CD16^+^ monocytes in RA patients was restricted to the CD14^+^CD16^+^ (and not CD14^dim^CD16^+^) monocyte subset (Fig. 1B and 2B). The level of CD14^dim^CD16^+^ was not significantly changed in RA patients, indicating that CD14^dim^CD16^+^ cells were not responsible for the expansion of CD16^+^ monocytes in RA (Fig. 1B and 2B). The CD14^dim^CD16^+^ monocyte subset was reported to be a functional homolog of the *patrolling* murine LY6C^low^ (Gr1^−^) monocyte subset. These cells have an important role in innate local surveillance of tissues and in antiviral immunity through sensing of viral DNA. The CD14^dim^CD16^+^ monocyte subset produces a greater amount of both the IL-1 receptor antagonist (IL-1Ra), which prevents IL-1-mediated signaling responses by competing with IL-1, and the IL-10 receptor [4]. Thus, in this context, CD14^dim^CD16^+^ monocytes appear to have an anti-inflammatory role, if any, and are not likely involved in the pathogenesis of RA. In contrast, CD14^+^CD16^+^ monocytes are the most potent producers of TNF-α, IL-1β, and IL-6 in response to various stimuli. Thus, expanded CD14^+^CD16^+^ monocytes in RA patients play a central role contributing to the pathogenic environment of inflamed joints.

Co-signaling molecule networks among immune cells are critical for controlling the immune response [17]. Among these, the B7 superfamily is major co-signaling molecules acting as a checkpoint in the modulation of T-cell responses [23]. Given that *in vivo* activated-monocytes derived from synovial fluid of active RA patients promote pathogenic Th17 responses through cell contact, it was suggested that differential expression of co-signaling molecules might be responsible. Here we show the upregulation of CD80 (B7-1) and CD276 (B7–H3), which are members of B7 superfamily, on synovial monocytes and not on peripheral monocytes in RA patients (Fig. 3). Both CD80 and CD276 are known to be inducibly expressed on monocytes under stimulation conditions, whereas CD86 (B7-2) is constitutively expressed on resting monocytes. As CD80 and CD276 were found to be induced on monocytes by TLR or cytokine stimulation, our data suggest that synovial monocytes, but not peripheral monocytes, from RA patients are in an activated state, presumably due to the surrounding cytokine milieu [24], [25]. Although induction of CD276 is closely linked to activation and differentiation of monocytes, its exact functional role is still controversial [26]. Whether CD276-mediated signaling in T cells plays a pathogenic role in RA remains to be elucidated.

Trafficking of CD16^+^ monocytes is directly dependent on the CX_3_CR1- CX_3_CL1 interaction under both steady-state and inflammatory conditions [6], [27], [28]. Therefore, it is reasonable to assume that CX_3_CR1 would be more highly expressed on synovial monocytes than on peripheral monocytes, if the increase in synovial CD14^+^CD16^+^ monocytes observed here is due to selective migration from the periphery; however, the expression of CX_3_CR1 mRNA was found to be decreased considerably in synovial monocytes of RA patients (Fig. 4A). More interestingly, CX_3_CR1 expression on synovial monocytes was very similar among the three monocyte subsets (Fig. 4D). This implies that CD14^+^CD16^−^ monocytes may be recruited into inflamed joints and gain expression of the CD16 molecule under inflammatory conditions present within synovial fluid. To this point, it should be noted that the majority of CD14^+^CD16^+^ monocytes in the mucosa of intestinal bowel disease (IBD) patients are derived from CD14^+^CD16^−^ cells, and these cells undergo a phenotypic switch to CD16 expression under inflammatory conditions [14].

Thus, the key question is how synovial monocytes gain this unique phenotypic feature in inflamed joints of RA. Since CD14^+^CD16^+^ monocytes are markedly expanded in cancer patients repeatedly treated with LPS and in sepsis patients, it has been suggested that the increase in cytokines caused by LPS or septicemia contribute to the induction of CD16 on monocytes [29], [30]. Indeed, CD16 expression can be induced by certain cytokines found in inflammatory conditions such as TGF-β, IL-10, and M-CSF [31]. In this study, we showed that CD16 expression on monocytes was significantly elevated following treatment of cells with TGF-β, an effect that was successfully inhibited by a specific blocker of TGF-β signaling, SB431542 (Fig. 6C). However, major pro-inflammatory cytokines such as TNF-α, IL-1β, and IL-6, which are abundant in inflamed joints, did not positively influence CD16 induction on monocytes (Fig. 6D). In spite of the role of TGF-β for CD16 induction, our ELISA showed that the average amount of TGF-β in the plasma of healthy controls was comparable to that in RA patients (data not shown). TGF-β mainly exists in a latent form and the amount of active TGF-β in most of biological samples is below the detection limit using conventional ELISA. Since TGF-β activation locally occurs at the cell surface, local concentration of bioactive TGF-β is presumably more important for their biological functions than total amount of latent TGF-β. In this context, it should be noted that purified CD14^+^CD16^−^ monocyte aggregated with activated platelet, one of major source of bioactive TGF-β, markedly induced CD16 with TGF-β signaling dependent manner [32]. The interplay between TGF-β and monocytes is responsible for amplification of monocyte-mediated inflammatory responses in an autocrine fashion. TGF-β is produced by monocytes at sites of inflammation and TGF-β-stimulated monocytes release an inactive, latent form of TGF-β that in turn, is activated by monocytes [33], [34]. In the present study, the expression of CD80 and HLA-DR on synovial monocytes (Fig. 3) was also predominantly enhanced by IFN-γ, which is abundant in inflamed joints and probably released by Th1 cells [35]. Given that HLA-DR and CD80 play a critical role for delivering ‘signal 1’ and ‘signal 2’ to T cells, respectively, synovial monocytes could have immunostimulatory effects on T cell responses at the site of inflammation.

Activated monocytes exert fundamental effects on the polarization and expansion of T cells and can contribute to shaping primary and secondary T-cell responses [7]. Thus, synovial monocytes with distinct functional phenotypes can modulate T-cell responses in RA. Recently, it was reported that Th17 responses are selectively promoted by *in vivo*-activated synovial monocytes derived from active RA patients. In addition, Rossol et al. showed that LPS-treated CD14^+^CD16^+^ monocytes preferentially promote the expansion of the Th17, but not Th1, subset and none of the monocyte subsets influence Th1 expansion in healthy donors [36]. However, our findings clearly show that synovial monocytes, and not peripheral monocytes, derived from RA patients markedly facilitate production of IFN-γ as well as IL-17 from autologous, peripheral CD4^+^ memory T cells (Fig. 8). It is unclear why not only Th17, but also Th1 subsets are significantly expanded in our experimental system. Perhaps differences in culture systems utilized by us and others are responsible for this disparate result. Another possible cause is our use of RA patient-derived CD4 memory T cells and not total CD4 T cells. In the clinical setting, CD4 T cells, which have a chance to associate with activated monocytes in the inflamed joints, are likely to be memory T cells due to the inability of naive T cells to be recruited into the site of inflammation. Indeed, a majority of synovial T cells belong to the memory subset (data not shown). Although Th17 cells undeniably play a critical role in RA pathogenesis, it should be noted that in RA patients a large proportion of synovial CD4^+^ T cells are also IFN-γ-producing Th1 cells [35]. It is therefore possible that synovial monocytes provide the recruited CD4 memory T cells with a favorable environment for the enhancement of IFN-γ and IL-17 production in the inflamed joints.

In summary, our study reveals the phenotypic and functional characteristics of monocytes in the synovial fluid and peripheral blood of RA patients, which may be affected by the synovial fluid environment and thus, critically affect the pathogenesis of RA. Thus, this suggests that the synovial fluid cytokine milieu imparts unique features upon synovial monocytes and thus, affects their critical roles in inflammatory T-cell responses during RA.
